# Sensor Information Fusion by Integrated AI to Control Public Emotion in a Cyber-Physical Environment

**DOI:** 10.3390/s18113767

**Published:** 2018-11-04

**Authors:** Seul-Gi Choi, Sung-Bae Cho

**Affiliations:** Graduate Program in Cognitive Science, Department of Computer Science, Yonsei University, Seoul 03722, Korea; abooun@yonsei.ac.kr

**Keywords:** cyber-physical system, sensor information fusion, emotion processing, Bayesian networks, reinforcement learning

## Abstract

The cyber-physical system (CPS) is a next-generation smart system that combines computing with physical space. It has been applied in various fields because the uncertainty of the physical world can be ideally controlled using cyber technology. In terms of environmental control, studies have been conducted to enhance the effectiveness of the service by inducing ideal emotions in the service space. This paper proposes a CPS control system for inducing emotion based on multiple sensors. The CPS can expand the constrained environmental sensors of the physical space variously by combining the virtual space with the physical space. The cyber space is constructed in a Unity 3D space that can be experienced through virtual reality devices. We collect the temperature, humidity, dust concentration, and current emotion in the physical space as an environmental control elements, and the control illumination, color temperature, video, sound and volume in the cyber space. The proposed system consists of an emotion prediction module using modular Bayesian networks and an optimal stimulus decision module for deriving the predicted emotion to the target emotion based on utility theory and reinforcement learning. To verify the system, the performance is evaluated using the data collected from real situations.

## 1. Introduction

As networks are increasingly opened up to wider access, the cyber-physical system (CPS), which is a next-generation smart system combining computing with a physical space, has been actively studied. The CPS determines and controls the physical space based on the sensor data collected. Sensor data serve as the eyes and ears to monitor the physical space, and controlling the space based on judgments made in the virtual space serves as the hands. It has become important in diverse domains as the uncertainty of the physical space can be ideally controlled using cyber technology [1]. CPS applications include energy management, traffic volume management, and healthcare systems.

In particular, it is necessary to tailor a service environment that can enhance customer satisfaction in the service space. In the space, there are physical aspects, such as various spatial factors, to be considered, but the emotions of the customer are also important. The correlation between the customer’s emotions and the effect of the service has been revealed through many studies. For example, students’ emotions may affect learning and achievement, and those of customers may be a decisive factor in purchasing [2].

**Figure 1:**
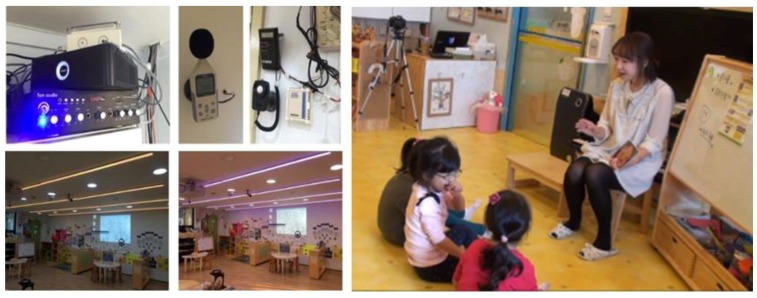
Emotion control experiment in a kindergarten. Left four photos show the sensors attached at the kindergarten, and right one shows a classroom with one teacher and three children.

However, to control emotions in the space, it is first necessary to recognize them. Various modalities, such as facial expressions and speech, have been applied for emotion recognition, but it is difficult for a person to wear equipment to collect data for such recognition. We have previously conducted a study to recognize and control emotions based on environmental stimuli in the space to induce target emotions [3]. This approach can overcome the constraint of wearing it in the space. Figure 1 shows the sensors and teaching scene in a kindergarten.

**Figure 2:**
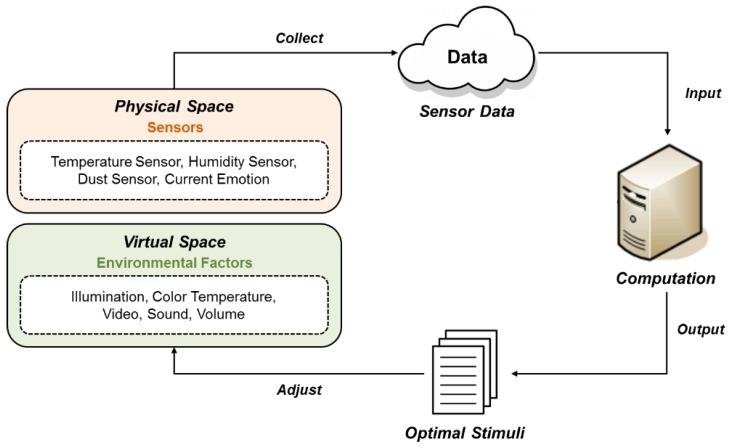
Overview of the proposed system.

To apply emotions to services through environmental stimuli in various domains, it is necessary to verify them for each domain. For example, the same illumination can cause various emotions depending on the space. It is also necessary to search for the optimal combination of stimuli to induce a specific emotion for each domain. In this paper, we propose a CPS control system for inducing emotions based on environmental sensors as shown in Figure 2. The virtual space is a 3D model that implements a real physical space and can be experienced by wearing VR equipment. It can address constraints on environmental stimuli of the physical space. A new stimulus can be provided without limitations on its kind and value.

In the CPS, the emotion inducement system consists of two parts. First, emotions are predicted using modular Bayesian networks based on the environmental sensors. The environmental stimuli are temperature, humidity, dust, and current emotion in the physical space, and illuminance, color temperature, video, volume, and sound in the virtual space. The optimal stimuli are then determined using utility theory to induce the predicted emotion to the target emotion. After reflecting the determined stimuli into the environment, feedback is given to them through reinforcement learning. To verify the proposed system, we implemented a commercial facility using Unity3D and conducted the experiment with various scenarios, and examined the possibility of the system for emotion inducement through a quantitative and qualitative analysis of the experimental results.

The remainder of this paper is organized as follows: Section 2 reviews the background of CPS and emotion recognition; in Section 3, the architecture and description of the proposed system are presented; in Section 4, we demonstrate the performance of the proposed system and compare it with conventional models; and in Section 5, some conclusions as well as the future works are discussed.

## 2. Related Works

### 2.1. Cyber-Physical System

**Figure 3:**
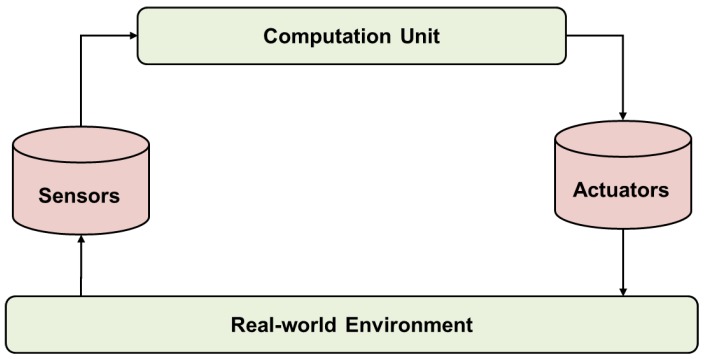
Components of the cyber-physical system.

The CPS consists of sensors and actuators in physical space and a computation unit in virtual space as shown in Figure 3. The virtual space of the CPS is different from the virtual space mentioned above, which is the 3D model. The log data monitored in real time through sensors in the physical space are processed in the computation unit of the virtual space, and then the physical space is controlled by the actuator [4]. Based on low-level sensor data collected in the physical space, it is possible to extract high-level information such as recognition and prediction in a virtual space. With the development of the network, applications can be made in various domains regardless of the size of the domain.

There are many researchers working on the CPS as shown in Table 1. Longhi et al. have installed sensors in waste containers to manage solid waste in the city [5]. A waste management system based on the amount of waste monitored through the sensors was proposed. Wan et al. proposed a CPS for the energy management framework based on the amount of energy collected from the navigation of an autonomous electric vehicle in a smart grid [6]. Zhang et al. suggested a smart health system which stores and analyzes large amounts of information such as environmental sensors, biosensors, and personal information collected from cameras in the healthcare domain [7]. To secure the operator hoisting the girder in the offshore, Han monitored the location information through the smartphone and built an alarm system based on it [8]. Jia et al. proposed a tracking method using the Wi-Fi of a smartphone rather than GPS or Bluetooth to track the user’s behavior [9].

This paper proposes a CPS control system for emotion services from environmental sensors. It collects temperature, humidity, and dust information from sensors in the physical space, and stores illumination, color temperature, video, sound, and sound information in the virtual space. After the environment information is transmitted to the server, the current emotions are predicted, and the optimal stimuli are determined to induce the predicted emotion into the target emotion, and reflected to the virtual-physical space through the actuators.

### 2.2. Emotion Recognition

Emotions affect human thinking and behavior. There have been attempts to recognize them in various aspects according to their importance. The modality is the information used for the emotion recognition, such as facial expression, speech, bio-signal, and text. Table 2 shows the relevant work on emotion recognition.

Facial expression and speech are the most direct emotional information, and Kudiri et al. acquired them in dialogue to recognize eight basic emotions (anger, sadness, happiness, boredom, disgust, fear, surprise, and neutral) using support vector machine (SVM) [11]. Wollmer et al. recognized the valence and activation by applying bi-directional long short-term memory (BLSTM) to them in the video [12]. Gunes et al. proposed a multimodality-based emotion recognition method that recognizes emotions based on them, respectively, and integrates these emotions [13]. For each modality, six emotions (happiness, fear, anger, anxiety, contempt, and uncertainty) were classified using the Bayesian network. In addition, Wagner et al. recognized four emotions (pleasure, anger, joy, and sorrow) through k-nearest neighbor (kNN), linear discriminant function (LDF), and multi-layer perceptron (MLP) based on electromyogram (EMG), electrocardiogram (ECG), skin conductivity (SC), and respiration (RSP) after inducing the emotions through music [14]. Soleymani et al. recognized three emotions (calm, positive excited, and negative excited) using the Bayesian network composed of expert knowledge with visual scene information, auditory sound information, and the title of the movie [15].

The above studies are aimed at collecting individual data and recognizing the individual’s emotions. However, people with the same environmental stimulus within the same space form a similar emotion. This can be inferred based on the environmental information by analyzing the psychological influence of each stimulus.

**Figure 4:**
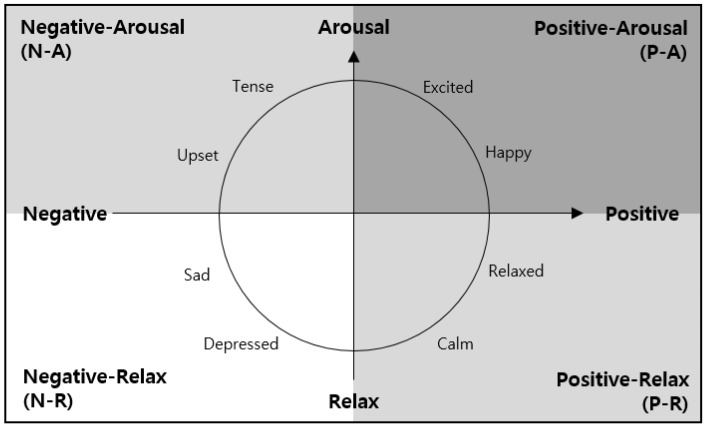
The circumplex model of emotion (redrawn from Reference [16]).

In this paper, we use sensor data in the virtual-physical space as a modality. A Bayesian network, which is a probability model, is applied considering the uncertainty of sensor data and the complexity of emotion. We model the psychological influence of the environmental stimuli in the space to predict emotions. The predicted emotions are classified as positive-arousal (P-A), negative-arousal (N-A), negative-relax (N-R), and positive-relax (P-R) by applying Russell’s circumplex model as shown in Figure 4 [16].

## 3. The Proposed System

### 3.1. System Overview

**Figure 5:**
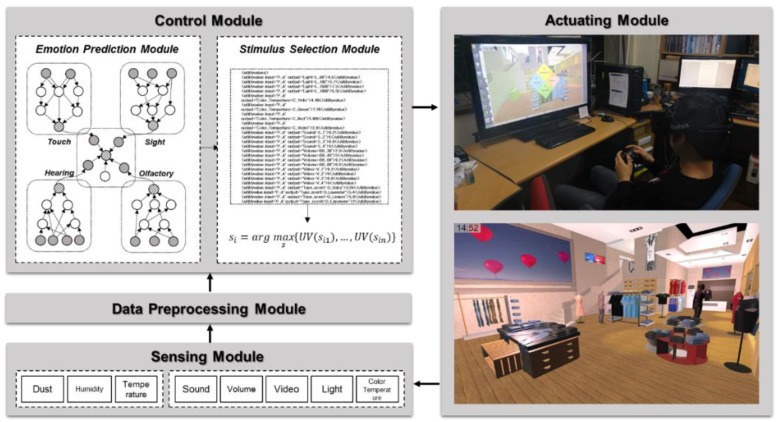
The proposed system.

This paper proposes a virtual-physical control system for emotion inducement from environmental sensors. Sensors and actuators to adjust emotions are installed in a virtual-physical space. The data collected through sensors are transmitted to the server to predict the current emotion, and then the optimal stimuli to induce the predicted emotion to the target emotion are determined. The selected stimuli are delivered to the actuators to control the environment. Figure 5 shows the overview of the proposed system.

#### 3.1.1. System Architecture

In the emotion inducement system, the virtual space is a 3D graphic model implemented by Unity3D, and the physical space is the experimental space. Users wear VR equipment in the physical space to experience the virtual space. Within the physical space, tactile, olfactory, and auditory information are provided, and visual information is in the virtual space. Sensors and actuators are installed in each space. Table 3 describes the definition of the virtual-physical space.

The proposed system includes a data collection server and an optimal stimulus decision server. The data collection server collects and stores environmental information for the virtual-physical space. The optimal stimulus decision server preprocesses the sensor data collected, predicts the emotions, and determines the optimal stimuli to induce the target emotion. Each server is composed of modules, with specific functions, connected through communication modules.

The environment measurement and control of the spaces consist of the following three modules:

- Physical space measurement module: Temperature, humidity and dust information are collected through sensors installed in the physical environment. They are collected in real time, 1 sec.
- Virtual space control module: Color temperature, video, volume, and sound information output in the virtual space. It outputs in real time, 20 sec.
- Current emotion input module: In the physical space, the controller receives the current emotion of the user. It receives input in real time, 20 sec.

The emotion control system of the virtual space also consists of the following three modules:

- Data pre-processing module: The sensor data collected in the virtual-physical space is preprocessed with the discretized values using the pre-processing rules.
- Emotion prediction module: Based on the discretized sensor information, the emotions are predicted through the modular Bayesian networks.
- Stimulus selection module: The optimal environmental stimuli are determined to induce the predicted emotion to the target emotion. The optimal stimuli are selected using the utility value of each stimulus which is defined in the utility table.

**Figure 6:**
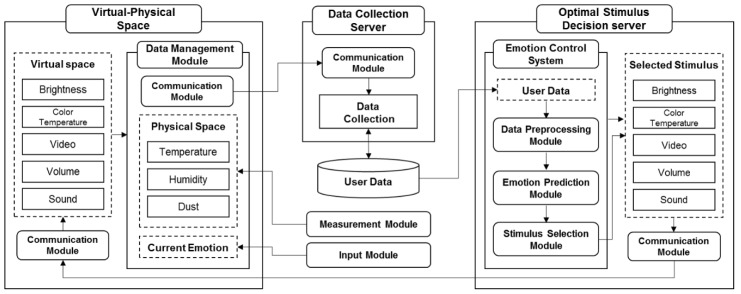
System architecture.

Figure 6 shows the communication among the modules in the virtual-physical space.

#### 3.1.2. System Specifications

The emotion inducement system requires specific hardware and software. For a computer to run software, in particular, the same or greater specific specifications are required.

Hardware can be divided into input and output devices. We used Microsoft’s Xbox 360 controller as the input device for the user who can experience the virtual space through the controller, and enter the user’s emotion using the up, down, left, and right buttons on the controller. The input devices for collecting environmental stimuli are dust concentration, humidity and temperature sensors, which are attached to Arduino UNO R3.

**Figure 7:**
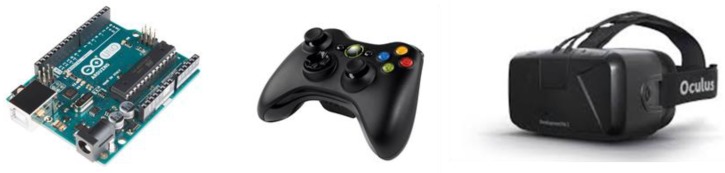
Hardware (input devices and output device): Arduino UNO R3, Microsoft Xbox 360, and Oculus VR DK2 from left to right.

Software can be divided into a program for configuring a virtual environment and a program for a physical environment. We need the Unity program for modeling the virtual environment in 3D graphics, the Oculus program for supporting the controller and the head mount display (HMD). Arduino IDE is also required to receive the sensor data using the Arduino board. The specific specifications for the programs are given in Table 4, and Figure 7 shows the hardware devices used in this paper.

#### 3.1.3. System Configuration

**Figure 8:**
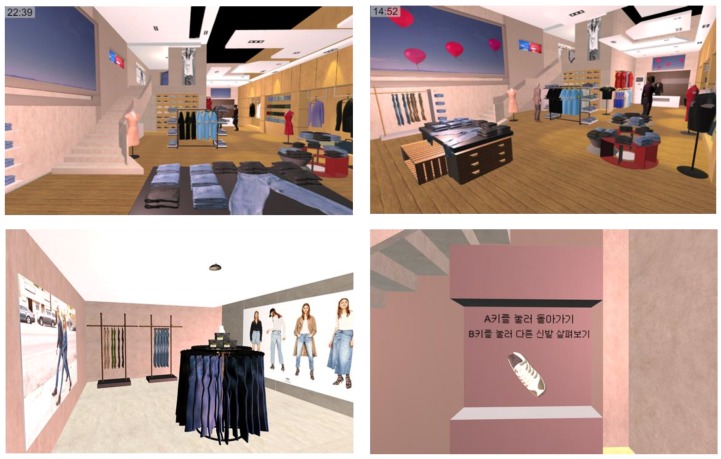
Virtual space (Commercial facility). First three photos show the virtual space in different angles, and the last photo shows a shoe to be chosen by the user.

In the virtual-physical space, the emotion control system predicts the emotions based on the environmental stimuli, and adjusts the stimulus to induce the predicted emotion to the target emotion. We built a virtual environment by designing a commercial facility as a 3D model for the virtual-physical space. It is a store in two floors, with reference to actual stores. There are clothes, accessories, shoes, etc. in the store, and virtual people who are employees and guests. The virtual environment is shown in Figure 8.

The environmental stimuli controlled in the virtual-physical space are shown in Table 5. In the physical space, temperature, humidity, dust, and current emotion are controlled, and in the virtual space, illumination, color temperature, video, volume, and sound are controlled. The values of each stimulus are discretized for the input of the emotion prediction model. The temperature, humidity, and dust concentration are divided into four states within the range that can be collected in the indoor space. Current emotion is entered as one of four values (positive-arousal, negative-arousal, negative-relax, and positive-relax) based on Russell’s emotion model [16]. In the case of illumination, color temperature, video, volume, and sound, the values are selected as the stimuli for each of the four emotions. The stimulus value set in the virtual space is presented in Table 6.

### 3.2. Emotion Control System

**Figure 9:**
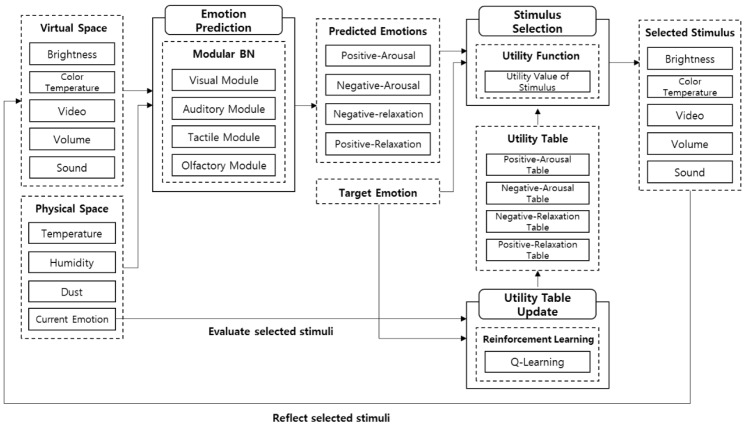
Emotion control system.

The emotion control system consists of an emotion prediction module and a stimulus decision module. The emotion prediction module predicts emotions based on environmental information. The stimulus decision module determines the optimal stimuli to drive the predicted emotion to the target emotion. The process of the system is shown in Figure 9.

#### 3.2.1. Emotion Prediction Model

The emotion prediction model predicts the emotions affected by space based on the sensor data of the virtual-physical space. The model can be defined as follows:

(1) $$\;f:\left\{C,\;E_{T}\right\}\to E_{T\;+\;1}$$

where $E_{T}$ is the current emotion state, $E_{T\;+\;1}$ is the predicted emotion state, and $C=\left\{S_{1,}S_{2,}\ldots ,S_{N}\right\}$ is *N* kinds of environmental stimuli.

The emotion prediction model needs to consider two points. First, the sensor data collected in the real environment is incomplete and uncertain [20]. Uncertainty must be managed to ensure the performance of the system using the sensor data [21]. Second, there is the complexity of emotion. There are infinite kinds of emotions, and their variety can be felt at one point.

We applied a Bayesian network as the emotion prediction model. It can effectively manage the uncertainty through probabilistic reasoning. The Bayesian network is defined as B= 〈G,θ〉, where G is a directed acyclic graph (DAG) and θ is a parameter set constituting the network. The graph consists of nodes $X_{1},\;X_{2},\ldots ,X_{n}$, which are variables, and arcs representing the connection relationship between the nodes. Each node has a conditional probability table (CPT), and when the set of parent nodes for node X is pa(X), it is obtained from Equation (2) based on the chain rule.

(2) $$\;P\left(X\right)=\;P(X_{1},X_{2,\;}\ldots X_{n})=\;P(X_{1}),P\left(X_{2}|X_{1}\right),\ldots ,\;P\left(X_{n}|X_{1},X_{2,\;}\ldots X_{n-1}\right)\;\;=\;\overset{n}{\underset{i=1}{\prod }}P(X_{i}|pa\left(X_{i}\right))$$

Since the Bayesian network can be designed using domain knowledge, it can be used even if there are not enough data in the actual application. To design the network, psychological influences of environmental stimuli on the emotions are investigated through literature. Based on the domain knowledge, we design the emotion inference model with environmental stimuli in space. The input and output of the emotion prediction model are described in Table 7.

We modularize the network by function to predict emotions in various domains. The emotion prediction model consists of a general module, a domain selection module, and a domain-specific module.

- General module: It is used regardless of domain. It consists of the elements that can be applied to all domains.
- Domain-specific module: It can be used only in a specific domain. Based on the previous study, each domain has sensory (visual, tactile, olfactory, and auditory) modules [3].
- Domain selection module: It connects general modules with domain-specific modules. It determines the specialized module to use according to the domain.

**Figure 10:**
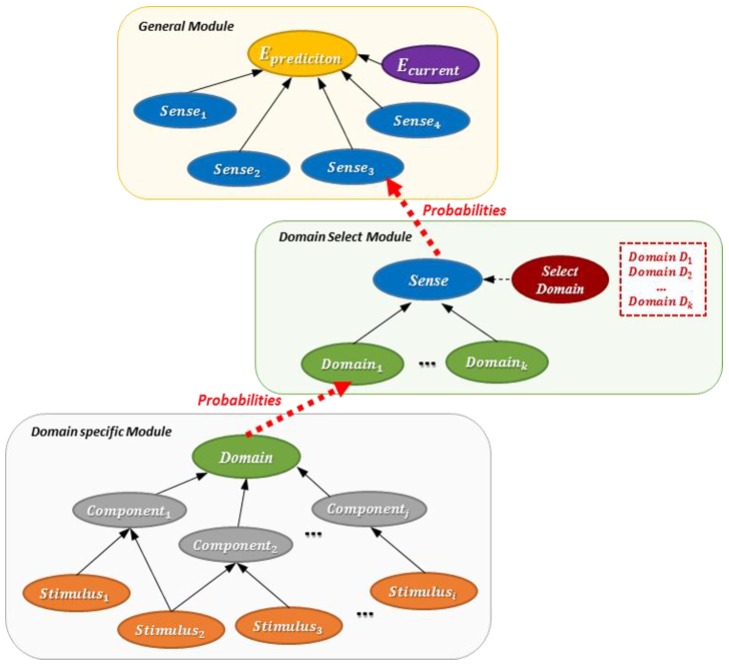
Emotion prediction model.

The structure of the model is shown in Figure 10. There are 17 modules in total, one general module, four domain selection modules, and 12 domain-specific modules. The module information is summarized in Table 8.

#### 3.2.2. Stimulus Decision Algorithm

The Bayesian network for predicting the emotion outputs the probabilities for the four emotions as a result. The proposed system finds appropriate stimuli to induce the target emotion based on the probability of these emotions. The target emotion is the ideal emotion for the service at this moment. The emotion is set to one of P-A, N-A, N-R, and P-R.

The proper stimulus is determined using a utility function that calculates utility values in utility tables, which defines the utility value indicating the influence of the stimulus on the target emotion in the predicted emotion. The utility value in the table is expressed as UV (Stimulus, Predicted Emotion, and Target Emotion). To determine the optimal stimuli, the influence of each stimulus calculates an expected utility value EU considering all four predicted emotions for the target emotion. The expected utility value $EU_{s}^{e_{t}}$ of each stimulus s for the target emotion $e_{t}$ is calculated using Equation (3).

(3) $$\;EU_{s}^{e_{t}}=\underset{e_{p}\in E}{\sum }UT\left(s,\;e_{t},\;e_{p}\right)\cdot prob_{e_{p}}\;$$

where $prob_{e_{p}}$ is the predicted probability of emotion, and $UT\left(s,e_{t},e_{P}\right)$ is the utility value of the stimulus.

To induce the target emotion, we found out what state each stimulus should have through the expected utility value. The set of states in each stimulus g is $S_{g}=\left\{s_{1},s_{2},\ldots ,s_{n}\right\}$, given the set of adjustable stimuli in Table 9 as$\;G=\left\{g_{1},g_{2},\ldots ,g_{m}\right\}$. The optimal stimulus state for stimulus g is determined by $Opt_{s}^{g}$ having the maximum expected utility value.

(4) $$\;Opt_{s}^{g}=\arg\;\underset{s}{\max}\left\{EU\left(s_{1}\right),EU\left(s_{2}\right),\ldots ,EU\left(s_{n}\right)\right\}\;$$

When the optimal stimulus state for all the stimuli is determined, the environmental stimulus is adjusted to the optimal state to the current environment to induce the target emotion.

**Figure 11:**
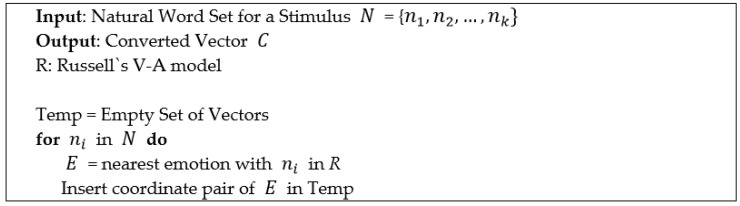
Natural word conversion algorithm.

**Initializing Utility Table:** The proposed system uses the domain knowledge to initialize the utility table. The initialization process consists of two rules. The first rule is to convert the domain knowledge provided in natural language into a two-dimensional numeric vector. One dimension represents the degree of valence, and the second dimension represents the degree of arousal. We used Russell’s V-A model to obtain the degree of each [16]. The detailed procedure is shown in Figure 11. The second rule maps the two-dimensional vector obtained in the first rule to the utility table. The value of the utility table is set according to the following equation based on the transformed vector $C=\left\{c_{v},c_{a}\right\}$ for each stimulus:

(5) $$\;UT\left(s,e_{PA},e_{P}\right)=c_{v}+c_{a}+b\;UT\left(s,e_{NA},e_{P}\right)=-c_{v}+c_{a}+b\;UT\left(s,e_{NR},e_{P}\right)=-c_{v}-c_{a}+b\;UT\left(s,e_{PR},e_{P}\right)=c_{v}-c_{a}+b\;$$

where *b* is the basis of the utility table.

**Updating the Utility Table**: Utility tables initialized with domain knowledge may not be perfect, because there may be a difference between the domain knowledge and the actual environment. The proposed system updates the table using Q-learning, one of the reinforcement learning methods. It uses Q-value $Q_{t,s}$ to indicate the fitness of stimulus *s* in the environment at time *t*, and finds the parameters to maximize the Q-value. The Q-value is updated with the reward, and the reward at *t* $r_{t}\left(e_{target},e_{t}\right)$ is determined using Equation (6).

(6) $$r_{t}\left(e_{target},e_{t}\right)=\;\{\begin{array}{c} R\;if\;e_{target}\;and\;e_{t}\;completely\;matches\\ \;0\;if\;e_{target}\;and\;e_{t}\;partially\;matches\\ -R\;if\;e_{target}\;and\;e_{t}\;completely\;different \end{array}$$

where *R* is the constant reward of the system.

In the proposed system, the Q-value is defined as the utility value in the utility table. This means that $Q_{t,s}\left(e_{\mathrm{target}},e_{t}\right)$ is the same as $\mathrm{UT}\left(s,e_{t},e_{P}\right)$ at *t*. Then, updating the Q-value means updating the utility value of the utility table. The update formula of the Q-value is shown in Equation (7).

(7) $$\;Q_{t+1,s}\left(e_{target},e_{t}\right)=\left(1-\alpha \right)\;Q_{t,s}\left(e_{target},e_{t}\right)+\alpha r_{t}\left(e_{target},e_{t}\right)\;$$

where α is the updating ratio of the Q-value.

## 4. Experiments

We have designed several scenarios for the virtual-physical space to verify the proposed system. Based on the scenarios, we evaluated the accuracy of the emotion prediction model and the performance of the stimulus decision algorithm.

### 4.1. Experiment Design

#### 4.1.1. Scenario Design

**Emotion Scenario:** Emotion scenarios should include all emotion change cases to verify the influence of environmental stimuli on the changes. The emotion change cases are shown in Table 10. They can be divided into four types: positive-negative, negative-positive, arousal-relax, and relax-arousal. The scenarios are designed to reflect all change cases based on possible situations in a real store.

**Behavior Analysis**: To design the emotion scenarios, we analyzed possible behaviors in shopping stores. Based on the theory of Leont’ev, an activity is analyzed by subject, object, action, and operation [22]. The analyzed behaviors are shown in Table 11.

- Subject: The actor engaged in the activity. In the physical space, there is a store employee who is an experimenter and a guest who is a subject. Virtual persons (employees and guests) are also randomly placed in the virtual space.
- Object: The objects are derived by a subject with a specific intention. They include clothes, accessaries, and shoes in the store.
- Action: A goal-oriented act must be undertaken to achieve the subject’s intention. In total, ten behaviors are analyzed.
- Operation: The phenomenon of the subject that appears irrespective of the intention.

**Designed Scenario**: Emotion scenarios are designed to include all emotional change cases based on analyzed behaviors. The scenario time is set in consideration of the subject’s concentration and fatigue. We designed five scenarios, taking a total of 29 min. They consisted of a variety of shopping situations (interaction with employees, product view, purchase and refund, etc.). The scenarios are presented in Table 12, Table 13, Table 14, Table 15 and Table 16.

#### 4.1.2. Experimental Data

Experiments were conducted based on the designed scenarios to verify the proposed system. Ten undergraduates and graduate students from Yonsei University attended (seven males and three females). For each scenario, the emotion was measured every 20 sec, and then the optimal stimuli to induce the target emotion were presented. The emotion was input through the up, down, left, and right buttons on the controller that matched the representative emotions of positive-arousal, negative-arousal, negative-relax, and positive-relax. The subject pressed the button of the emotion that is most similar to the current emotion among the four buttons in the guidance text of “Please input emotion” on the screen presented at intervals of 20 sec.

**Figure 12:**
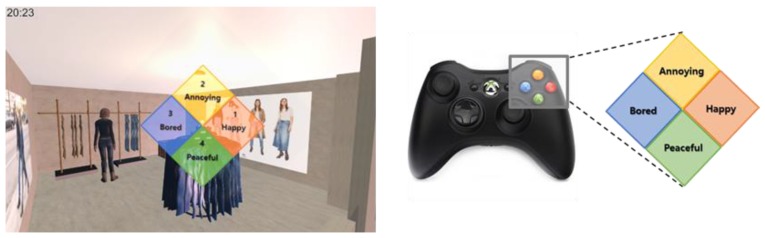
Emotion collection screen (**left**) and emotion input button (**right**).

Figure 12 shows the emotion collection screen and buttons for emotion collection. The total number of collected data is 858. The distribution of data is shown in Table 17.

### 4.2. Result

#### 4.2.1. Emotion Prediction Model

The performance of the emotion prediction model for a four-class problem is evaluated with prediction accuracy, which is compared with the predicted emotion at time t and the current emotion at time t+1. False-positive and false-negative errors might provide a full assessment of the performance, but the main focus of this research was on adjusting emotion in a subjective way that is less affected by objective accuracy. In any case, we undertook a comparison with other classification techniques (DT, MLP, kNN, and SVM) for a fair evaluation. 

**Figure 13:**
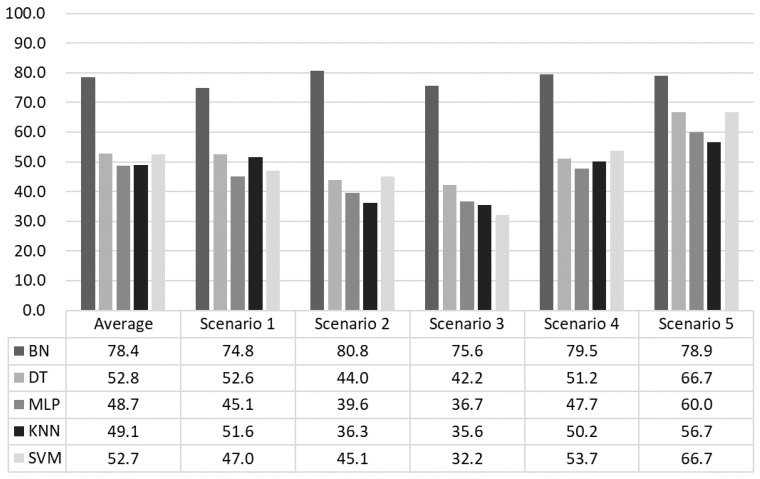
Emotion prediction accuracy.

As can be seen in Figure 13, the proposed BN outperforms the alternatives unquestionably. The experimental results show that the accuracy of the proposed model was 78.4%, which was about 27.6% higher than the average accuracy of 50.81% for the others. In particular, scenario 2 had the highest accuracy and scenario 1 had the lowest accuracy. This is because scenario 2 had only positive-negative changes that are more likely to be induced than arousal-relax changes. Moreover, since scenario 1 is the least restrictive, the behavior patterns of the subjects varied. The current emotions measured by these behaviors were more varied than the other scenarios. Scenario 5 showed that all the methods performed better than other scenarios. It was the case that led to a negative situation as a whole, and it seemed that the subjects were fully immersed and induced to the target emotion.

#### 4.2.2. Stimulus Decision Algorithm

**Figure 14:**
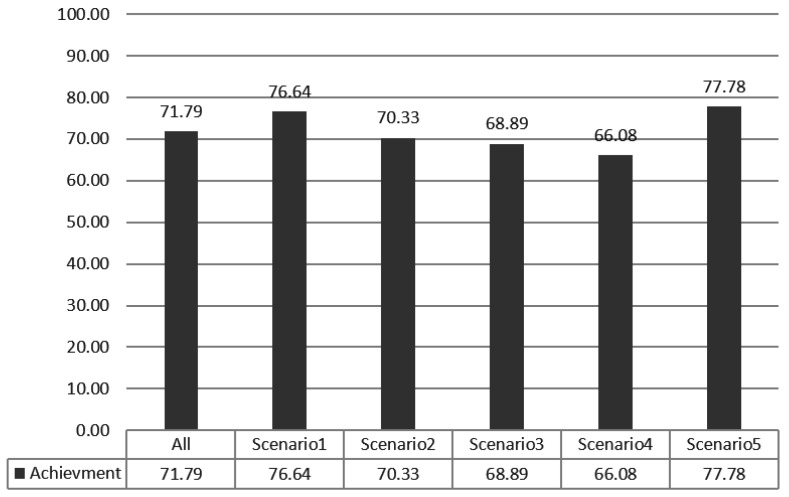
The results of emotion achievement for the five scenarios. The achievement depends on how similar the induced emotion is to the target emotion.

**Figure 15:**
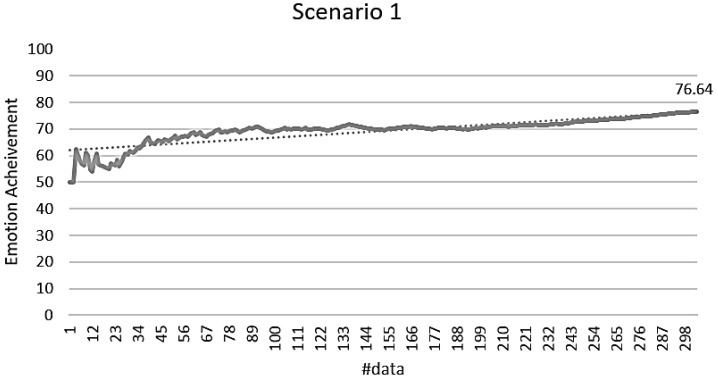
Emotion achievement for scenario 1. The thick line shows the increase of the achievement according to the number of sensory changes, and the dotted line shows the trend of this increase.

**Figure 16:**
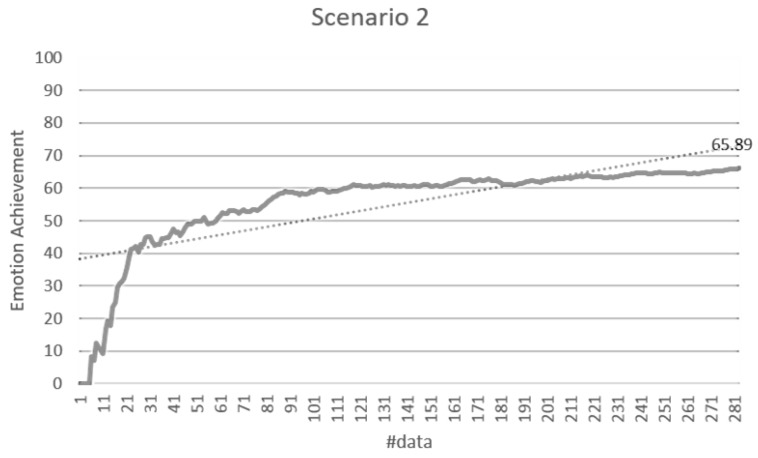
Emotion achievement for scenario 2. The thick line shows the increase of the achievement according to the number of sensory changes, and the dotted line shows the trend of this increase.

**Figure 17:**
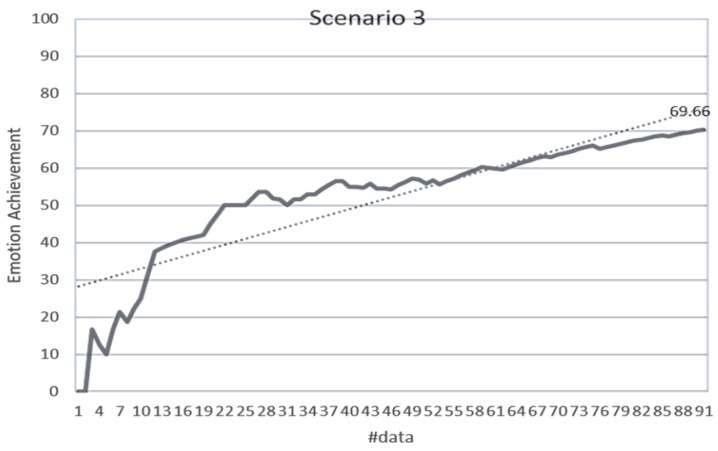
Emotion achievement for scenario 3. The thick line shows the increase of the achievement according to the number of sensory changes, and the dotted line shows the trend of this increase.

**Figure 18:**
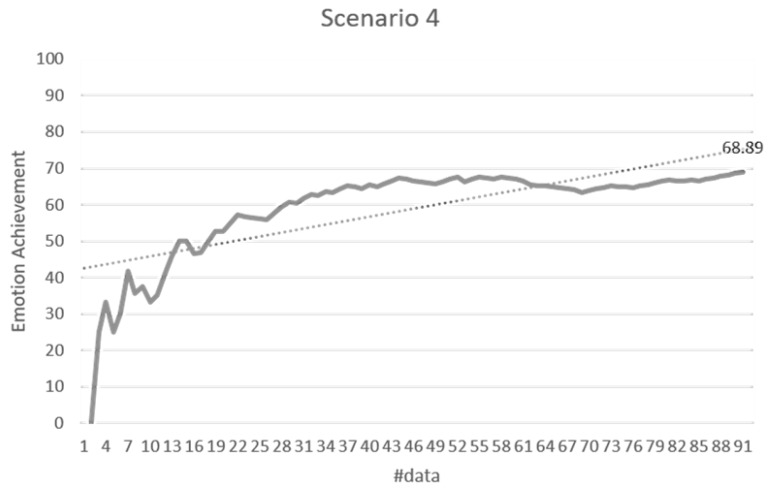
Emotion achievement for scenario 4. The thick line shows the increase of the achievement according to the number of sensory changes, and the dotted line shows the trend of this increase.

**Figure 19:**
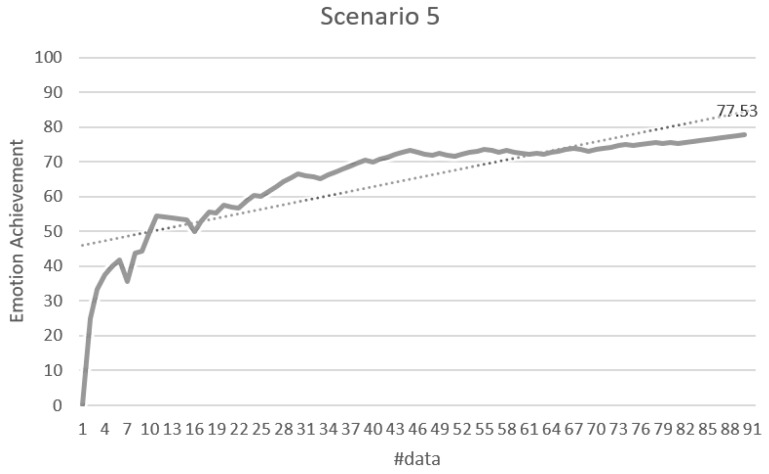
Emotion achievement for scenario 5. The thick line shows the increase of the achievement according to the number of sensory changes, and the dotted line shows the trend of this increase.

The validation of the stimulus decision algorithm was evaluated as the attainment of the target emotion. The achievement depends on whether the current emotion collected after the optimal stimuli determined to induce the target emotion is consistent with the target emotion. It indicates how much the determined stimuli have induced the target emotion. If the target emotion and the current emotion are the same, the achievement is 100%. If both emotions are the same as either positive-negative or arousal-relax, it is 50%, and if both are different, it is 0%. The experimental results in Figure 14 showed 71.79% for the total data. Scenario 1 was 76.64%, scenario 2 was 70.33%, scenario 3 was 68.89%, scenario 4 was 66.08%, and scenario 5 was 77.78%. Figure 15, Figure 16, Figure 17, Figure 18 and Figure 19 show the emotion achievements for the five scenarios.

The analysis of the achievements by scenario shows that the achievement improves as the training data accumulate in all the scenarios. As the utility value is updated to fit the domain, the optimized value for each stimulus can be obtained to derive the target emotion. However, in all scenarios except scenario 1, the initial achievement started at 0%. This was the case where the output stimuli did not induce the initial target emotion set in the scenario, and it was found that the influence of the stimuli was insufficient for the subjects at the beginning of the experiment.

## 5. Conclusions

In this paper, we have proposed an emotion inducement system to the public based on environmental sensors in a virtual-physical space. Because of the infringement of privacy, we could not deal with individual emotions to control. As an application of CPS, it can be applied in various service spaces to enhance the effectiveness of the service. However, before its application, it is necessary to verify the influence of environmental stimuli and the optimal stimuli for each domain. To solve this problem, we built a virtual space that is an extension of the physical space and overcomes the constraints on the stimuli of the physical environment. The virtual space is a shopping store implemented as a 3D model, and users can experience it through VR equipment for immersion. The environmental stimuli to control emotions are temperature, humidity, and dust in the physical space, and illumination, color temperature, video, volume, and sound in the virtual space. 

The emotion control system first predicts emotions using modular Bayesian networks based on environmental sensors. The emotion prediction model showed that the performance was higher than with other classification methods. Then, the optimal stimulus is determined based on the stimulus decision algorithm to induce the predicted emotion to the target emotion. The utility value of the stimulus is defined as domain knowledge and stored in the utility table, and the optimal stimulus is searched through the expected utility value. However, since the output optimal stimuli may not induce the target emotion, we evaluate whether it induces the emotion, and its utility value is updated through reinforcement learning. The proposed algorithm showed that the output stimuli in all scenarios led to the target emotion with a similar performance. According to the results of emotion induction by scenarios, it was found that the emotion achievement was improved through reinforcement learning as the data accumulate. It is possible to obtain the optimal utility value of each environmental stimulus to induce the target emotion in various domains.

Future works could be pursued in the three directions: First, we need to increase the number of possible participants in the CPS. To do that, we need to develop a separate server to run the proposed system with a larger number of participants. Second, the virtual environment should be improved significantly to reflect the real situations as closely as possible to give the participants more immersive experience. Lastly, we need to evaluate the performance with more subjects in various different environments. In this case, the input method of the target emotion should be changeable to see what would happen if the user accidentally pressed the wrong button.

## Figures and Tables

**Table 1:** Summary of related work.

Author	Sensor	Actuator	Domain
Longhi [5]	Sensors built into the trash can	Waste management	Solid waste management
Wan [6]	Autonomous electric vehicle’s navigation	Energy management	Energy management
Zhang [7]	Environment sensors, Biosensors, Camera	Healthcare applications services	Healthcare
Han [8]	Smart phones	Alarming system	Hoisting monitoring
Jia [9]	Wi-Fi	Passenger flow statistics, Precision marketing, Criminal hunting	Behavior tracking
Hu [10]	Smart phones	Dynamic route choices	Traffic prediction

**Table 2:** Related work on emotion recognition.

Author	Modality	Method	Emotion Classification
Kudiri [11]	Facial Expression, Speech	SVM	8 classes (anger, sadness, happiness, boredom, disgust, fear, surprise, neutral)
Wollmer [12]	Facial Expression, Speech	BLSTM, HMM, SVM	2 classes (valence, activation)
Chen [17]	Facial Expression, Speech, Body Gestures	SVM, RF, LR	7 classes (anger, sadness, happiness, disgust, fear, neutral)
Gunes [13]	Facial Expression, Body Gestures	BN	6 classes (disgust, happiness, fear, anger, anxiety, uncertainty)
Wagner [14]	EMG, ECG, SC, PSP	kNN, LDF, MLP	4 classes (anger, sadness, joy, pleasure)
Ko [18]	EEG	BN	5 classes (joy, anger, neutral, sadness, surprise)
Alm [19]	Text	NLP	2 classes (positive, negative)
Soleymani [15]	Video, audio, text	BN	3 classes (calm, positive excited, negative excited)

**Table 3:** Definition of virtual-physical space.

Type	Definition	Description
Physical space	Experimental space	Tactile, Olfactory, Auditory information
Virtual space	3D graphic space	Visual information

**Table 4:** System specifications.

Type		Category	Content	Device
H/W	Input device	User	Controller	Microsoft Xbox 360
			Current emotion	Enter button on controller
			Temperature, humidity, dust	Arduino UNO R3
	Output Device	Environmental Stimulus	Display	Oculus VR DK2
S/W	Unity	User	VR program	PC (GPU: NVIDIA GTX 970/AMD 290 CPU: Intel i5-4590 RAM: 8 GB OS: Windows 7 SP1 64 bit)
	Oculus S/W	Environmental Stimulus	Controller/HMD program	
	Arduino IDE		Sensing program	

**Table 5:** System configuration (for video and sound, stimuli 1, 2, 3, and 4 induce P-A, N-A, N-R, and P-R respectively).

Space	Environment Stimulus	Stimulus Value	Description
Physical Space	Temperature	18 °C, 23 °C, 25 °C, 28 °C	Current temperature in space
	Humidity	20%, 30%, 50%, 70%	Current humidity in space
	Dust	0.25, 0.5, 0.75, 1	Current dust concentration in space
	Current emotion	P-A, N-A, N-R, P-R	Current emotion state in space
Virtual Space	Illumination	60 lx, 100 lx, 1500 lx, 1800 lx	Illumination of light in space
	Color temperature	Yellow, Green, Red, Purple	Color of light in space
	Video	V_1, V_2, V_3, V_4	Content of video in space
	Volume	30 dB, 45 dB, 60 dB, 80 dB	Volume of sound in space
	Sound	S_1, S_2, S_3, S_4	Content of sound in space

**Table 6:** Environmental stimuli of the virtual-physical space.

Category	Positive-Arousal	Positive-Relax	Negative-Arousal	Negative-Relax
Illumination	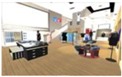	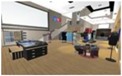	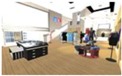	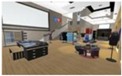
	1500 lux	100 lux	1800 lux	60 lux
Color temperature	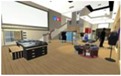	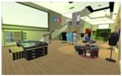	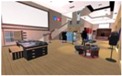	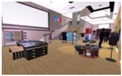
	Yellow	Green	Red	Purple
Video	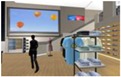	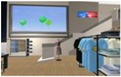	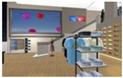	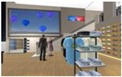
	V1(P-A)	V2(N-A)	V3(N-R)	V4(N-A)
Volume	45 dB	60 dB	80 dB	30 dB
Sound	S1(P-A)	S2(N-A)	S3(N-R)	S4(N-A)

**Table 7:** Input and output of emotion prediction model.

Type	Category	Element	#
Input	Physical Space	Temperature, Humidity, Dust, Current emotion	5
	Cyber Space	Illumination, Color temperature, Video, Sound, Volume	5
Output	-	Predicted Emotion	1

**Table 8:** Modules of emotion prediction model.

Type	Category	Input	Output	#	Description
General module	Emotion prediction module	Outputs of sensory modules, Current emotion	Predicted emotions	1	Predict final emotions based on four sensory modules
Domain selection module	Sensory modules	Outputs of sensory modules by domain, Domain information	Predicted emotions	4	Only reflect the results of that domain
Domain-specific module	Visual module	Illumination, Color temperature	Predicted emotions	3	Predict emotions based on visual stimuli by domain
	Olfactory module	Scent	Predicted emotions	3	Predict emotions based on olfactory stimuli by domain
	Tactile module	Temperature Humidity	Predicted emotions	3	Predict emotions based on tactile stimuli by domain
	Auditory module	Sound, Volume	Predicted emotions	3	Predict emotions based on auditory stimuli by domain

**Table 9:** Input and output of stimulus decision algorithm.

Type	Element	#
Input	Predicted emotions, Target emotion	2
Output	Illumination, Color temperature, Video, Sound, Volume	5

**Table 10:** Emotion change cases.

Case Number	Current Emotion	Target Emotion	Positive-Negative	Arousal-Relax
Case 1	1	1	Maintained	Maintained
Case 2		2	Positive → Negative	Maintained
Case 3		3	Positive → Negative	Relax → Arousal
Case 4		4	Maintained	Relax → Arousal
Case 5	2	1	Negative → Positive	Maintained
Case 6		2	Maintained	Maintained
Case 7		3	Maintained	Relax → Arousal
Case 8		4	Negative → Positive	Relax → Arousal
Case 9	3	1	Negative → Positive	Arousal → Relax
Case 10		2	Maintained	Arousal → Relax
Case 11		3	Maintained	Maintained
Case 12		4	Negative → Positive	Maintained
Case 13	4	1	Maintained	Arousal → Relax
Case 14		2	Positive → Negative	Arousal → Relax
Case 15		3	Positive → Negative	Maintained
Case 16		4	Maintained	Maintained

**Table 11:** Analysis of behaviors in a store.

#	Action	Subject	Object
1	Greet	Staff	-
2	Shop tour	Customer	-
3	Request product	Customer	-
4	Find product	Staff	Product
5	Recommend product	Staff	Product
6	Request recommendation	Customer	-
7	Reject product	Customer	Product
8	Like product	Customer	Product
9	Buy product	Customer	Product
10	Leave	Customer	-

**Table 12:** Scenario 1.

Time	1 min	2 min	3 min	4 min	5 min	6 min	7 min	8 min	9 min	10 min
Overall goal	Shopping									
Detailed goal	Entrance	Greeting	Guide products			Browse products		Induce to buy	Decide	Leave
Target emotion	P-R	P-A	P-A			P-R		N-A	P-R	P-R

**Table 13:** Scenario 2.

Time	0 min	1 min	2 min	3 min
Overall goal	Situations where you need something, but want nothing			
Detailed goal	You are excited	Entrance & Greeting	Induce to buy	Leave
Target emotion	P-A	P-A	N-A	P-A

**Table 14:** Scenario 3.

Time	0 min	1 min	2 min	3 min
Overall goal	Situations where you are tired and need something, but want nothing			
Detailed goal	You are tired	Entrance & Greeting	Induce to buy	Leave
Target emotion	N-R	P-A	N-A	P-A

**Table 15:** Scenario 4.

Time	0 min	1 min	2 min	3 min	4 min	5 min	6 min	7 min	8 min	9 min	10 min
Overall goal	Situations where you had an unpleasant experience, but you come to buy what you wanted										
Detailed goal	You are unpleasant	Entrance	Greeting	Guide products		Wait products		Recommend another product		Induce to buy	Leave
Target emotion	N-A	P-A	P-A	P-A		N-A		N-R		N-A	P-R

**Table 16:** Scenario 5.

Time	0 min	1 min	2 min	3 min
Overall goal	Refund			
Detailed goal	You are unpleasant	Entrance & greeting	Reject refund	Leave
Target emotion	N-A	N-R	N-A	N-R

**Table 17:** The number of data by emotion.

	Positive-Arousal	Negative-Arousal	Negative-Relax	Negative-Relax
#data	172	208	220	258
